# Diagnostic delay stages and pre-diagnostic treatment in patients with suspected rheumatic diseases before special care consultation: results of a multicenter-based study

**DOI:** 10.1007/s00296-022-05223-z

**Published:** 2022-10-10

**Authors:** Franziska Fuchs, Harriet Morf, Jacob Mohn, Felix Mühlensiepen, Yuriy Ignatyev, Daniela Bohr, Elizabeth Araujo, Christina Bergmann, David Simon, Arnd Kleyer, Wolfgang Vorbrüggen, Andreas Ramming, Jörg H. W. Distler, Peter Bartz-Bazzanella, Georg Schett, Martin Welcker, Axel J. Hueber, Johannes Knitza

**Affiliations:** 1 Department of Internal Medicine 3, Friedrich-Alexander-University Erlangen-Nürnberg (FAU) and Universitätsklinikum Erlangen, Ulmenweg 18, 91054 Erlangen, Germany; 2 Deutsches Zentrum Immuntherapie (DZI), Friedrich-Alexander-University Erlangen-Nürnberg and Universitätsklinikum Erlangen, Erlangen, Germany; 3 Faculty of Health Sciences, Center for Health Services Research, Brandenburg Medical School Theodor Fontane, Rüdersdorf, Neuruppin, Germany; 4 Verein Zur Förderung Der Rheumatologie E.V, Würselen, Germany; 5 RheumaDatenRhePort (rhadar), Planegg, Germany; 6 Klinik Für Internistische Rheumatologie, Rhein-Maas Klinikum, Würselen, Germany; 7 MVZ Für Rheumatologie Dr. Martin Welcker GmbH, Planegg, Germany; 8 Division of Rheumatology, Paracelsus Medical University, Klinikum Nürnberg, Nuremberg, Germany; 9 Section Rheumatology, Sozialstiftung Bamberg, Bamberg, Germany

**Keywords:** Diagnostic delay, Delayed diagnosis, Triage, Outcome research, Time to therapy, Health service research

## Abstract

Early and effective discrimination (triage) of patients with inflammatory rheumatic diseases (IRD) and other diseases (non-IRD) is essential for successful treatment and preventing damage. The aim of this study was to investigate diagnostic delays and pre-diagnosis treatment in patients newly presenting to rheumatology outpatient clinics. A total of 600 patients newly presenting to one university hospital and two non-academic centers were included. Time from onset of symptoms to rheumatology consultation “total delay” as well as medical treatment before consultation were recorded. Median time from symptom onset to rheumatologist appointment (total delay) was 30 weeks. Median time to online search, first physician appointment request and first physician appointment was 2, 4 and 5 weeks, respectively. Total delay was significantly shorter for IRD patients compared to non-IRD patients, 26 vs 35 weeks (*p* = 0.007). Only 17.7% of all patients and 22.9% of IRD patients had a delay of less than 12 weeks. Total delay was significantly lower in patients seen in non-academic centers compared to the university center, 20 vs 50 weeks (*p* < 0.0001). 32.2% of IRD patients received medical treatment that eased their symptoms prior to the rheumatology appointment. These findings highlight the persistent diagnostic delays in rheumatology; however, they also suggest that current triage strategies effectively lead to earlier appointments for IRD patients. Improvement of triage methods and pre-diagnosis treatment could decrease overall burden of disease in IRD patients.

**Supplementary Information:**

The online version contains supplementary material available at 10.1007/s00296-022-05223-z.

## Introduction

Considerable evidence shows that early diagnosis and treatment leads to better outcomes in patients with inflammatory rheumatic diseases (IRD) [1–3] and that the first 12 weeks after symptom onset represent a therapeutic window of opportunity [4]. Patients with any joint swelling, associated with pain or stiffness, should be seen by a rheumatologist within 6 weeks according to the current European Alliance of Associations for Rheumatology (EULAR) recommendations for early arthritis [2]. The German Association for Rheumatology recommends that patients suspected to have an IRD should be seen by a rheumatologist within 2 weeks [5].

The currently declining number of rheumatologists and the increasing demand in consultations [6], impede the implementation of these ambitious goals in real life. Time between symptom onset and first rheumatologist appointment is often exceeding 12 weeks, more likely ranging between a median of 27 [7], 29 [8], 46 [9] and 120 [10] weeks. Similar to emergency care, where high patient demand meets limited resources, rheumatologists have to triage patients with the goal of reducing the time-to-therapy for IRD patients. Diagnostic delay is, however, also largely determined by factors related to patients, referring physicians and disease-related factors [11]. Furthermore, rheumatic patients often experience unspecific musculoskeletal symptoms, which are difficult to interpret for patients [7] and even rheumatologists [12].

On the other hand, patients and their environment are increasingly consulting online search engines (SE) [13, 14], online self-referral tools and symptom checkers [15, 16] before consulting a doctor to assess and interpret their symptoms. Due to the permanent availability and easy usage, effective digital symptom assessment and treatment recommendations could contribute to cutting down diagnostic delays and overall disease burden in rheumatology.

We could recently demonstrate that confined to basic health and symptom-related medical history, the diagnostic accuracy of rheumatologists was lower compared to an AI-based symptom checker [17]. Nearly half the patients presenting to rheumatologists used an online search engine prior to their appointment to assess symptoms [16]; however, it is unclear when patients looked up their symptoms and, therefore, how much diagnostic delay cut potentially be cut down. Similarly, other process indicators that contribute to a correct diagnosis and early treatment need to be analyzed to guide future process improvements.

To better understand the current situation, we analyzed different parts of diagnostic delay as well as pre-diagnosis treatment in patients, who were newly presenting to rheumatology outpatient clinics.

## Methods

This cross-sectional survey study recruited adult patients with unknown diagnosis newly referred to rheumatology outpatient clinics at one university hospital (University Clinic Erlangen) and two practices (MVZ für Rheumatologie Planegg and Sozialstiftung Bamberg) between September 2019 and April 2021. Clinical diagnosis was based on medical history, physical examination, laboratory and imaging results. No standard diagnostic or triage approach was predefined and local rheumatologists could freely decide on diagnostic investigations and time to see referred patients. Patients completed a questionnaire to collect information regarding four parts of diagnostic delay, including the time (1) until first online search to interpret symptoms; (2) until first contact to physician contacted to assess symptoms; (3) until first physician appointment and (4) until rheumatologist appointment. Patients also stated whether they received any medication for their symptoms (yes/no), and if such treatment eased their symptoms (yes/no).

This study was part of a randomized trial that primarily evaluated the diagnostic accuracy of two patient facing diagnostic decision support systems (DDSS). The methods and interim results of the diagnostic accuracy have been previously published [18]. The study was approved by the ethics committee of the Medical Faculty of the University of Erlangen-Nürnberg (106_19 Bc) and was conducted in compliance with the Declaration of Helsinki. A meeting abstract was presented at the German rheumatology conference in 2022 [19].

### Statistical analysis

To record the general peculiarities of the health services, all centres were divided into two groups (university clinic and practices). Descriptive characteristics were presented as median (Mdn) and interquartile range (IQR) and mean and standard deviation (SD) for continuous data and as absolute (n) and relative frequency (percent) for categorical data. To compare health services between clinic and practices, Mann–Whitney *U* test was used for continuous variables with non-normal distribution, while chi-square test with Yates’ correction for continuity was used to compare frequencies for categorical variables. The significance level was set at *p* ≤ 0.05. The analyses were performed using IBM SPSS Statistics V.20 Windows (SPSS Inc, Chicago, Illinois, USA) and Excel Windows (Microsoft GmbH, Unterschleißheim).

## Results

### Patient demographics

In total, 600 patients were recruited. Demographic characteristics are displayed in Table 1. 214/600 (35.7%) patients were diagnosed with an IRD. Rheumatoid arthritis was the most common IRD diagnosis (69/600; 11.5%). A similar proportion of IRD patients presented to the University clinics as to the practices (34.1 vs 38.2%). Mean age was 49.6 years and 418/600 (69.7%) patients were female.

**Table 1:** Patient demographics

Patients	Total *N* = 600	Academic *n* = 367	Private *n* = 233
Age, years, mean ± SD	49.6 ± 15.4	48.2 ± 15.8	51.9 ± 14.5
Female, *N* (%)	418 (69.7)	259 (70.6%)	159 (68.2%)
Diagnostic category			
Non IRD, *N* (%)	278 (46.3%)	242 (65.9%)	144(61.8%)
Undifferentiated arthritis	19 (3.2%)	9 (2.5%)	10 (4.3%)
Axial spondyloarthritis	31 (5.2%)	21 (5.7%)	10 (4.3%)
Inflammatory, other	7 (1.2%)	7 (1.9%)	0 (0%)
Connective tissue disease	22 (3.7%)	18 (4.9%)	4 (1.7%)
Peripheral spondyloarthritis	3 (0.5%)	3 (0.8%)	0 (0%)
Rheumatoid arthritis	69 (11.5%)	28 (7.6%)	41 (17.6%)
Vasculitis	8 (1.3%)	4 (1.1%)	4 (1.7%)
Psoriatic arthritis	31 (5.2%)	22 (6.0%)	9 (3.9%)
Polymyalgia rheumatica	16 (2.7%)	7 (1.9%)	9 (3.9%)
Degenerative causes	71 (11.8%)	45 (12.3%)	26 (11.2%)
Fibromyalgia	37 (6.2%)	14 (3.8%)	23 (9.9%)
Crystal arthropathy	8 (1.3%)	6 (1.6%)	2 (0.9%)
Time from symptom onset to first web search, weeks, median (IQR)/mean ± SD	2 (0.4–4.3) 3.1 ± 23.1	2 (0.4–5.7) 4.2 ± 29.4	1.43 (0.4–4.3) 1.4 ± 3.4
Time from symptom onset to first physician request, weeks, median (IQR)/mean ± SD	4 (2–10) 14 ± 38.6	4 (2–10 14.4 ± 43.5)	4 (1–12) 13.3 ± 29.4
Time from symptom onset to first doctors´ appointment, weeks, median (IQR)/mean ± SD	5 (2–12) 15.9 ± 40.2	5 (2–12) 15.1 ± 42.9	6 (2–14) 17.2 ± 35.5
Time from symptom onset to first rheumatologists´ appointment, weeks, median (IQR)/mean ± SD	30 (12–82.5) 87.5 ± 152.8	50 (20–105.5) 112.7 ± 176.8	20 (8–50) 47.7 ± 91.2
Tender joint count, mean ± SD	1.6 ± 3.6	1.2 ± 3.5	2.3 ± 3.5
Swollen joint count, mean ± SD	0.9 ± 2.4	0.4 ± 1.3	1.8 ± 3.3
Visual analog scale global, cm, mean ± SD	38.4 ± 28.3	44 ± 25.3	29.2 ± 30.6
Morning stiffness, min, mean ± SD	16.8 ± 28.8	16.7 ± 26.4	17.1 ± 32.3
Erythrocyte sedimentation rate, mm/h, mean ± SD	14 ± 15.5	14 ± 15.4	14 ± 15.6
C-reactive protein, mg/L, mean ± SD	0.7 ± 1.3	0.8 ± 1.1	0.7 ± 1.5
Rheumatoid factor positivity, *N* (%)	71 (13%)	48 (13.6%)	23 (11.8%)
Anti-citrullinated protein antibody positivity, *N* (%)	25 (4.7%)	16 (4.7%)	9 (4.7%)

### Stages of diagnostic delay

Patients assessed their symptoms using online search engines after a median of 2 (0.4–4.3) weeks (mean 3.1 ± 23.1), requested a first physician appointment after a median of 4 (2–10) weeks (mean 14 ± 38.6), had their first physician appointment after a median of 5 (2–12) weeks (mean 15.9 ± 40.2) and had their first rheumatologist appointment after a median of 30 (12–825) weeks (mean 87.5 ± 152.8), respectively (Table 2).

**Table 2:** Diagnostic delay stages and treatment according to different centers

	All Settings *n* = 600	University *n* = 367	Practice *n* = 233	W-Value/Χ^2^ Value	*P*-Value
Time from symptom onset to first web search, weeks, median (IQR)/mean ± SD	2 (0.4–4.3) 3.1 ± 23.1	2 (0.4–5.7) 4.2 ± 29.4	1.4 (0.4–4.3) 1.4 ± 3.4	65,130.500	0.009
Time from symptom onset to first physician request, weeks, median (IQR)/mean ± SD	4 (2–10) 14.0 ± 38.6	4 (2–10) 14.4 ± 43.5	4 (1–12) 13.3 ± 29.4	66,564.000	0.093
Time from symptom onset to first doctors´ appointment, weeks, median (IQR)/mean ± SD	5 (2–12) 15.9 ± 40.2	5 (2–12) 15.1 ± 42.9	6 (2–14) 17.2 ± 35.5	109,583.500	0.734
Time from symptom onset to rheumatologists´ appointment, weeks, median (IQR)/mean ± SD	30 (12–82.5) 87.5 ± 152.8	50 (20–105.5) 112.7 ± 176.8	20 (8–50) 47.7 ± 91.2	54,714.000	< 0.0001
Pre-consultation therapy received, *N* (%)	325 (49.8%)	224 (61.0%)	101 (43.3%)	17.25	< 0.0001
Pre-consultation therapy helped and symptoms improved, *N* (%)	151 (25.2%)	106 (28.9%)	45 (19.3%)	6.43	0.011

**Fig. 1:**
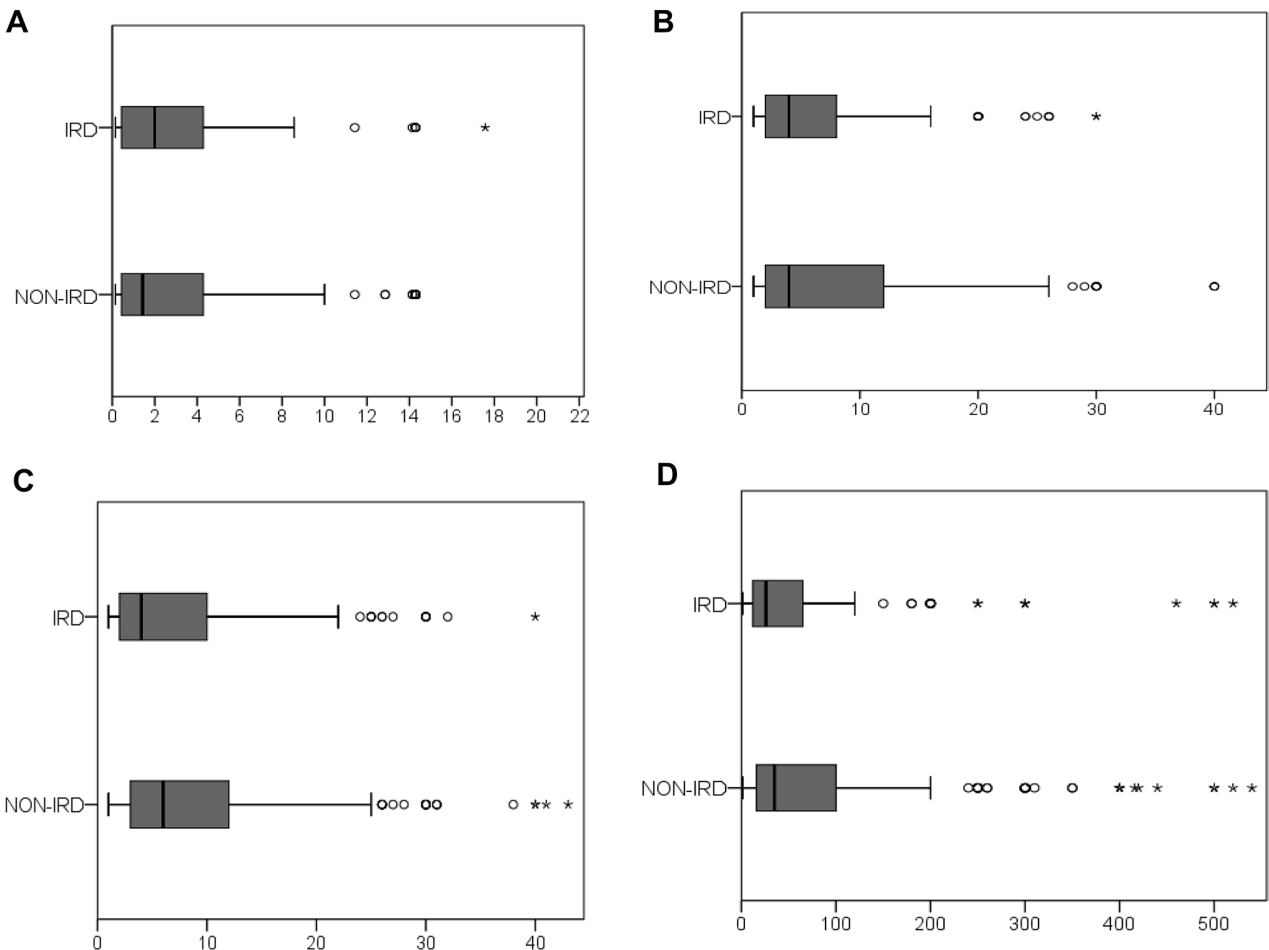
**A** Time from symptom onset to first web search (weeks); **B** time from symptom onset to first physician request (weeks); **C** time from symptom onset to first doctors´ appointment (weeks); **D** time from symptom onset to first rheumatologists´ appointment (weeks), according to inflammatory rheumatic disease (IRD; yes/no) status

Vasculitis patients reported the shortest total delay (mean: 24.3 ± 31.1 weeks; median of 7 (5–60)), while axSpA patients (mean: 153.2 ± 310.8 weeks; (median of 50 (24–78)) had the longest delay (Supplementary Material 1). IRD patients’ delay was significantly shorter in all analyzed stages as compared to non-IRD patients, with a median total delay of 26 weeks (12–66.5) vs. 35 weeks (15.5–100; *p* = 0.007) (Fig. 1).

**Fig. 2:**
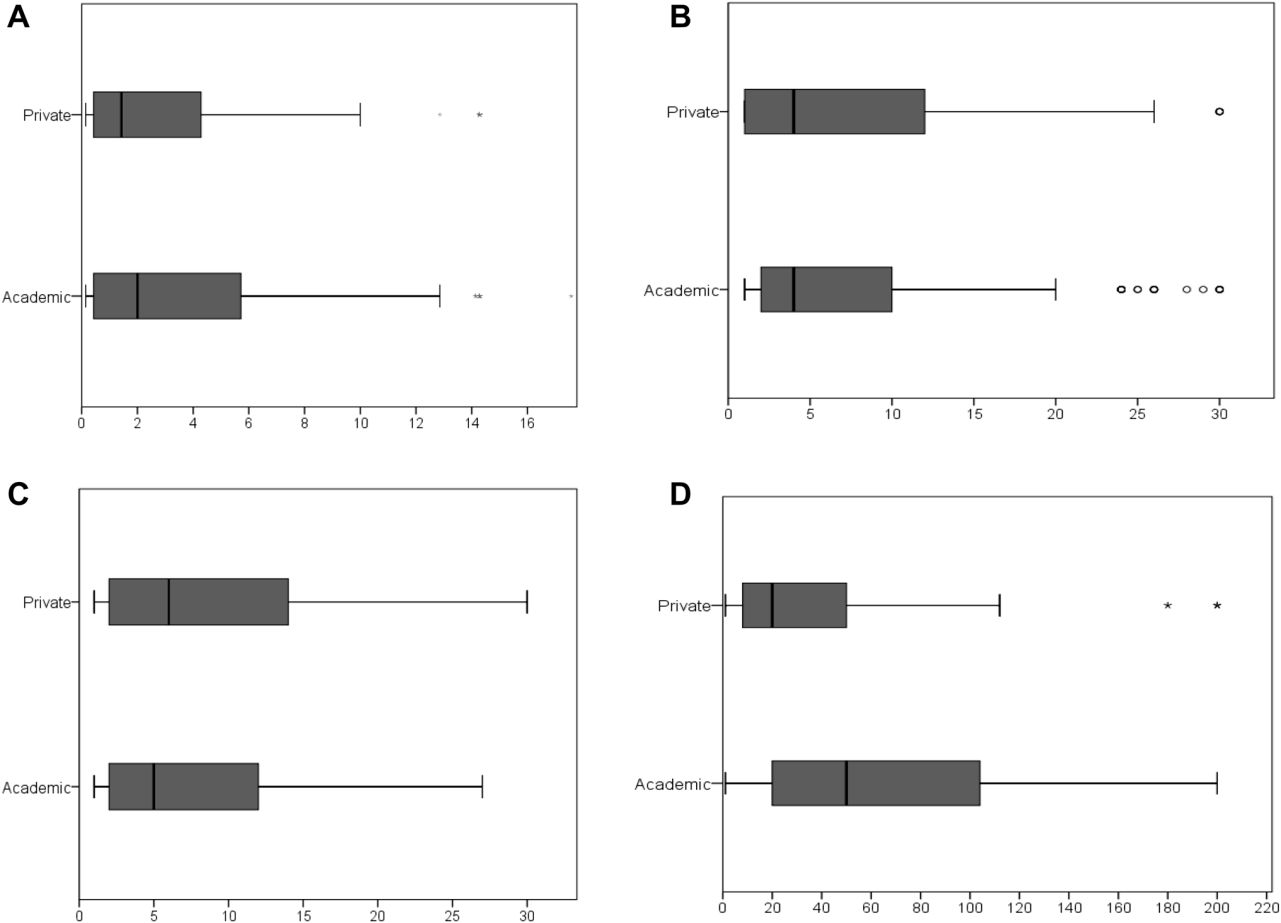
**A** Time from symptom onset to first web search (weeks); **B** time from symptom onset to first physician request (weeks); **C** time from symptom onset to first doctors´ appointment (weeks); **D** time from symptom onset to first rheumatologists´ appointment, according to university center/practice (weeks)

Patients seen at practices reported a significantly shorter median total delay compared to patients seen in the University center, (20 (8–50) weeks vs. 50 (20–105.5) weeks; (W = 54,714.000, df = 1, *p* < 0.0001), see Fig. 2. Only 49/214 (22.9%) of IRD patients and 57/386 (14.8%) of non-IRD patients had a total delay of less than 12 weeks.

### Pre-diagnostic treatment

325/600 (54.2%) patients received medication prior to the rheumatologist appointment (IRD: 57.5%; non-IRD 52.3%), see Table 2. 69/214 (32.2%) and 82/386 (21.2%) of IRD and non-IRD patients, respectively, received medication and also experienced a symptom improvement.

## Discussion

The aim of this study was to investigate the diagnostic delay stages and the pre-diagnostic treatment in patients newly presenting to German rheumatology outpatient clinics. We could show that total diagnostic delay is still a major challenge with a median duration of 30 weeks, which by far exceeds the often stressed “window of opportunity” of 12 weeks. Encouragingly though, diagnostic delay was significantly shorter in IRD patients at all four investigated stages compared to non-IRD patients. Similarly, our work highlights the importance of practices, as the total delay was significantly shorter in the practices than in the university centers. To our knowledge, this study is the first to investigate time from symptom onset until online search and to compare diagnostic delay between practices and university centers.

Our work demonstrates that diagnostic delay still represents a major challenge largely caused by lack of rheumatologists [5] and the substantial referrals of non-IRD patients. Interestingly, we could show that patients attending practices had a significantly shorter diagnostic delay compared to university centers, despite comparable proportions of IRD patients within the patients with musculoskeletal complaints, being in line with previous findings [20]. This situation does not necessarily mean very similar patient groups referred to clinics and practices but could also be due to the fact that patients with rarer and more diagnostically challenging diseases (i.e., connective tissue diseases) accounted for a larger proportion at the academic center compared to a larger proportion of rheumatoid arthritis patients in the practices. In contrast to emergency medicine [21], no standardized, transparent and objective triage system is implemented in rheumatology care.

The shortest diagnostic delay was reported by vasculitis and (other) inflammatory patients, mainly adult-onset Still’s disease. Elevated inflammatory markers in these patients could have facilitated and prompted early rheumatology referral. The longest total delay with a mean of 153 weeks was reported for axSpA patients. AxSpA being the IRD with one of the longest diagnostic delays is in line with previous results from Asia [9], Denmark [22] and Germany [8]. Redeker et al. did not observe a substantial difference in the diagnostic delay in axSpA between the 1996–2005 period and the 2006–2015 period and identified a negative HLA-B27 status as the most important factor associated with a longer diagnostic delay in axSpA [10]. Our observed delay, however, is shorter than that found in health insurance data from 1677 axSpA patients (5.7 years) [10]. This observation could suggest a recent trend towards reduction of diagnostic delay [22], which may be partially attributed to a broader usage of online search engines and symptom checkers among IRD patients [15, 16]. These tools have a great potential to further reduce diagnostic delay suggesting that remote care (telemedicine) should increasingly be adopted into clinical routine according to current recommendations [23]. Among the four stages of diagnostic delay analyzed, the time until the final rheumatologist appointment was by far the longest, highlighting rheumatologists as the main bottleneck. Krusche et al. recently investigated that the lack of rheumatologists will likely even worsen in the next decade in Germany [6]. We believe that increasing the number of rheumatologists should be the main priority to cut down diagnostic delay. Symptom checkers [18] and home-self sampling [24] can contribute to patient-centered care, by overcoming time and geographic limitations, providing patients and rheumatologists with the necessary information [12] to make better informed diagnostic decisions. For clear cases, i.e., CPP-positive patients with reported joint swelling, telemedicine could likely accelerate diagnosis. The high number of false-positive IRD suggestions by symptom checkers [18] could, however, also contribute to an even greater workload. In addition, structured involvement of trained health care personnel can liberate physician time and improve focused care [25].

Only a minority of IRD patients received medication that eased symptoms prior to their rheumatologist consultation. A major reason could be a selection bias, as patients free of complaints likely cancel appointments and remain with their primary physician. Stack et al. reported that patients that purchased over-the-counter medications took longer to seek help than those who did not [7].

There are a number of strengths and limitations of this study. The representative sample of newly referred patients being included in a prospective multicenter randomized controlled trial is a strength of the study; however, the recruitment of patients in only one country and a limited number of centers limit the generalizability of results. To our knowledge, time until online symptom assessment has not been evaluated before in rheumatology patients. Measurement of key performance indicators such as diagnostic delay should be mandatory for rheumatologists and be collected in a centralized way to enable value-based healthcare and performance comparisons and ultimately improvement of care in rheumatology across different countries. Furthermore, memory bias represents a limitation of this study, as the results are based on patient reported data and we did not record the specific types of treatment. Variables such as socioeconomic status, educational level, residence (rural or urban) and occupational status likely also influence diagnostic delay and were not collected.

## Conclusion

Although current triage strategies enable significantly shorter appointments for IRD patients, treatment and diagnostic delay is still too long. Diagnostic delay varies significantly according to type of disease and type of rheumatology center.

## Supplementary Information

Below is the link to the electronic supplementary material.

Supplementary file1 (DOC 46 KB)

## Data Availability

Data are available on reasonable request from the corresponding author on reasonable request.
