# Supplementary material for: Diagnostic delay stages and pre-diagnostic treatment in patients with suspected rheumatic diseases before special care consultation: results of a multicenter-based study
Source: Rheumatol Int. 2022 Oct 10;43(3):495–502. doi: 10.1007/s00296-022-05223-z (PMC9968271; doi:10.1007/s00296-022-05223-z)
Supplement: Supplementary file 1 — Supplementary file1 (DOC 46 KB) [file 296_2022_5223_MOESM1_ESM.doc]

**Supplementary Material 1**: Time from symptom onset until rheumatologist appointment (main delay) and treatment received prior to rheumatologist appointment.

| Diagnostic category | | N (%) | Main diagnostic delay,  weeks, median (IQR) / mean ± SD | Therapy received, N (%) |
| --- | --- | --- | --- | --- |
|  | NON-RMD | 278 (46.3) | 30 (13-80)  82.48 ± 137.2 | 136 (48.9) |
|  | Arthritis | 19 (3.2) | 16 (7-60)  35.4 ± 38.7 | 9 (47.4) |
|  | Axial spondyloarthritis | 31 (5.2) | 50 (24-78)  153.2 ± 310.8 | 23 (74.2) |
|  | Inflammatory, other | 7 (1.2) | 20 (6-27)  19.4 ± 17.0 | 4 (57.1) |
|  | Connective tissue disease | 22 (3.7) | 57.5 (18-200)  124.1 ± 152.1 | 9 (40.9) |
|  | Peripheral spondyloarthritis | 3 (0.5) | 30 (24-30)  41.3 ± 25.0 | 3 (100.0) |
|  | Rheumatoid arthritis | 69 (11.5) | 25 (11-53.5)  48.3 ± 90.9 | 35 (50.7) |
|  | Vasculitis | 8 (1.3) | 7 (5-60)  24.3 ± 31.1 | 7 (87.5) |
|  | Psoriatic arthritis | 31 (5.2) | 30 (12-100)  116.6 ± 190.5 | 17 (54.8) |
|  | Polymyalgia rheumatica | 16 (2.7) | 17 (10.5-36.5)  45.1 ± 76 | 12 (75.0) |
|  | Degenerative cause | 71 (11.8) | 50 (18-130)  116.2 ± 173.8 | 45 (63.4) |
|  | Fibromyalgia | 37 (6.2) | 80 (23-150)  127.2 ± 157.6 | 21 (56.8) |
|  | Crystal arthropathy | 8 (1.3) | 27.5 (12.8-87.5)  55.5 ± 65.4 | 4 (50.0) |
